# Quantifying the Comprehensive Characteristics of Inclusion-Induced Defects Using an Integrated Destructive and Non-Destructive Method

**DOI:** 10.3390/ma14061475

**Published:** 2021-03-17

**Authors:** Rongfei Juan, Min Wang, Junhe Lian, Chao Gu, Lanxin Li, Yanping Bao

**Affiliations:** 1 State Key Laboratory of Advanced Metallurgy, University of Science & Technology Beijing, Beijing 100083, China; g20189216@xs.ustb.edu.cn (R.J.); gu_chao2011@sina.com (C.G.); lilanxin@crscd.com.cn (L.L.); baoyp@ustb.edu.cn (Y.B.); 2 Advanced Manufacturing and Materials, Department of Mechanical Engineering, Aalto University, Puumiehenkuja 3, 02150 Espoo, Finland; 3 CRSC Research & Design Institute Group Co., Ltd., Beijing 100070, China

**Keywords:** scanning ultrasound microscopy (SUM), largest extreme value distribution (LEVD), automatic inclusion analyzer (AIA), inclusions, electron microscope

## Abstract

Driven by the continuous improvement of the mechanical properties, especially the fatigue property of the high-strength steels, it is particularly important to characterize the type, size, and distribution of inclusions and the critical inclusions in the steel matrix, as they are decisive for the fatigue life performance. This paper presents an integrated approach for the comprehensive characterization of the inclusions in metals by combining the advantages of destructive methods based on metallography and non-destructive testing methods using ultrasonic detection technology. The position and size of inclusions were obtained by scanning ultrasonic microscope, and the composition and micro-image of inclusions were further analyzed by scanning electron microscope. According to the results obtained by the proposed approach, the distribution laws of oxide inclusions and sulfide inclusions in the samples were statistically analyzed, and then the maximum distribution analysis method was used to predict the maximum inclusions. We compare the predicted size value with the value obtained by the characterization method to establish a certain corresponding relationship. The results show that large defects in metals can be accurately characterized by the proposed method, and the size of inclusions predicted by extreme value analysis is close to that of the scanning electron microscope. The integrated destructive and non-destructive method can reveal the in situ information of inclusions and give the possible relationship between inclusions and process and material properties.

## 1. Introduction

Metals, in particular steels, are one of the most commonly used materials for various industrial sectors including automotive, energy, civil engineering, etc. In the context of the construction in civil applications, the excellent mechanical properties of steels, such as strength, toughness, and particularly fatigue properties are expected and pursued. In many aerospace materials and power generation materials used in high-temperature metals, to achieve their mechanical, chemical, and other aspects of high-temperature properties, the control of microstructure is very important [1,2]. However, non-metallic inclusions break the continuity of the metal matrix and result in the starting point of fatigue failure due to the stress concentration at the junction of the inclusions and the matrix [3,4]. During the thermal and deformation processes such as hot rolling and quenching, the difference of thermal shrinkage properties and elastic constants between the inclusions and the metal matrix can easily lead to stress concentration and defects [3,4,5,6].

According to the length scales, two types of defects are typically defined: macro-defects and micro-defects. Macro-defects refer to cracks, scratches, slag inclusion, etc., which can be found in surface appearance inspection, as well as shrinkage holes and bubbles, etc., which can be found in low-power pickling inspection. Micro-defects of metals include mainly small-scale non-metallic inclusions, which can be seen under the light microscope. Compared with macro-inclusions, micro-inclusions pay a minor contribution to the initiation of the crack but affect the expansion of forging cracks [7]. In practical application, non-metallic inclusions—especially macro-inclusions—in the metal matrix are closely relevant with the fatigue cracks, seriously affecting the performance of metal. Therefore, a thorough understanding and reasonable control of the non-metallic inclusions in the metal matrix through characterizing their sizes, morphologies, chemical compositions, spatial distribution, etc., becomes essential for clean metal production [8,9]. To characterize the inclusions accurately concerning the complex morphologies and distribution in the metal matrix, many different methods are developed to obtain the representative characteristics of inclusions [10,11].

Traditional metallographic methods (MM) for inclusion characterization possess randomness in the selection of cross-section and field of view, and they cannot present the difference in the spatial distribution of inclusions [12]. The full-scale analysis [13] is based on the addition of a small current electrolysis sample surface, which is then combined with scanning electron microscopy (SEM) and energy-dispersive scanning (EDS) to observe the morphology of inclusions. It can only separate inclusions on the surface by electrolysis, but it cannot locate the internal defects accurately [14]. In addition, it is difficult to distinguish between inclusions and the casting cracks caused by them [15]. Therefore, more advanced methods are needed to characterize the internal cleanliness of the material and more importantly the location and characteristics of large and critical defects.

Ultrasonic testing technology is non-destructive, which can be used to test the spatial distribution of inclusions at different thicknesses without demanding surface pretreatment [16]. Ultrasonic detection technology also possesses good directionality and high resolution, which is not only useful to detect small defects but also effective to avoid the omission of large defects. Therefore, the high-frequency ultrasonic detection method has been widely used in defect detection in various engineering materials [17]. Among several ultrasonic techniques, scanning ultrasonic microscope (SUM) as a commonly used ultrasonic flaw detector (UFD) can detect inclusions in slightly larger metal volume; the detection results are more accurate, and the detection time is relatively short. Li et al. [18] proposed a particle size characterization method based on ultrasonic energy attenuation coefficient spectrum and support vector regression (SVR). A statistical model was established between the energy attenuation coefficient of the sensitive frequency band and the average particle size.

Despite the great advantages, SUM or ultrasonic detection technology in general also suffers from several drawbacks. The detection resolution is always inversely proportional to the penetration depth [19], which means that a more detailed and comprehensive characterization of the defects has to come with the scarification of the tested volume. More critically, SUM can only obtain the maximum contour of the inclusion in space, but it cannot obtain its composition; i.e., the method has no distinction between different types of defects. As demonstrated by Gu et al. [20], besides the size and distribution of inclusions, the composition of inclusions is also very important for fatigue properties of high-strength steels. In addition, missing the inclusions composition information could also lead to a misinterpretation of inclusions size when two inclusions are very close to each other and can easily be regarded as one larger inclusion. Therefore, for accurate and comprehensive quantification of the defect characteristics of materials, a combination of the destructive and non-destructive methods is needed to obtain detailed inclusions characteristics, such as the distribution of size, shape, and composition while keeping the statistical and representative data in a bulk especially with respect to the large and detrimental macro-defects.

In the present study, an integrated method for the comprehensive characterization of defects characteristics in high-strength steel is proposed based on the combination of the advantages of the destructive and non-destructive methods. The SUM method is used to detect the defects of material in a large bulk volume to be able to collect the critical information of the large macro-defects, which could be simply overlooked by the metallographic-based method. By combining these two methods, the proposed approach could provide comprehensive and enriched information for the characterization of defects in the materials and provide substantial inputs for the microstructure–property correlation. With data obtained by the proposed method, the study also aims to establish a predictive model for the possible presence of large inclusions in a large volume of steel through the finite field of view using only the metallographic-based method. According to this model, the inclusions in the finite field of view are connected, and the size of inclusions in the large volume material is predicted. The model has the potential to overcome the disadvantage of the metallographic measurement for being local and missing the statistical information such as the large macro-defects and to upscale the metallographic method to bulk materials. In the present study, the accuracy of this model is compared and validated by the results from the SUM measurement.

In the following, the integrated method for the characterization of the defect characteristics will be introduced first. The measurement starts with the ultrasonic detection method. According to the ultrasonic echo scanning signal and images, the statistical information of the defect size distribution in the bulk is obtained and the spatial coordinates of the location are taken, and the specimen is dissected [21,22]. Combined with the SEM observation, the internal defects of the material can be located, qualitatively and quantitatively analyzed, and the defects such as crack, shrinkage cavity, and segregation in the steel can also be effectively detected. Finally, the distribution of inclusions in steel could be verified and compared by using an automated inclusion analyzer (AIA), and the distribution function of inclusions in steel is established. The size of the maximum inclusions in the plane will be predicted and verified by using the extreme distribution analysis method [23].

## 2. Experimental Material and Methods

### 2.1. Experimental Materials

**Figure 1:**
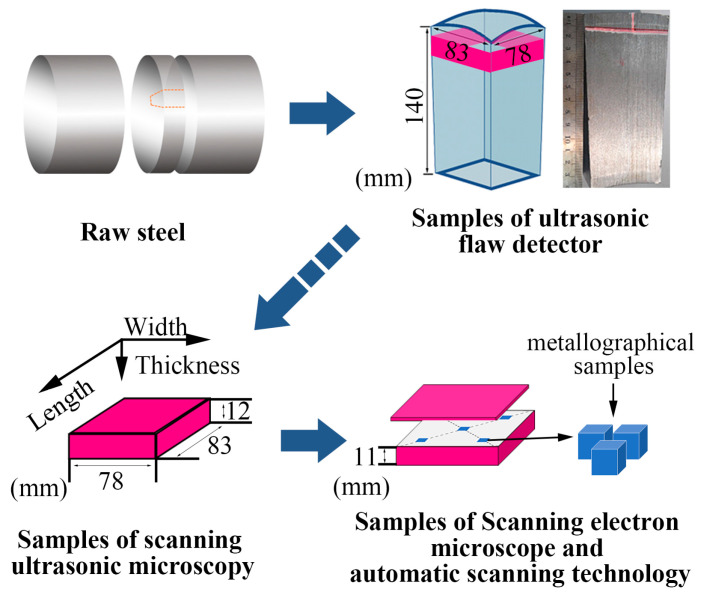
Experimental sample preparation process.

The chemical composition of the investigated steel is shown in Table 1 [24]. Figure 1 shows the sampling process and sample preparation. A cuboid with a cambered top of 104 mm × 83 mm × 78 mm was obtained by machining, and then ultrasonic inspection was carried out with a handheld ultrasonic flaw detection instrument. Then SUM was used to scan the 12 mm × 83 mm × 78 mm smooth block sample obtained by continuous processing.

SEM and an automatic inclusions scanning instrument were used to analyze the specific field of view, and the size of the sample processed was 10 mm × 10 mm × 10 mm. Most defects in the final steel product are closely related to inclusions. The characteristic of inclusions greatly affects the service performance and determines the fatigue life of the material. Thus, it is necessary to accurately characterize the distribution of inclusions, especially highly hazardous inclusions.

### 2.2. Ultrasonic Flaw Fetection and Scanning Ultrasound Microscopy

**Figure 2:**
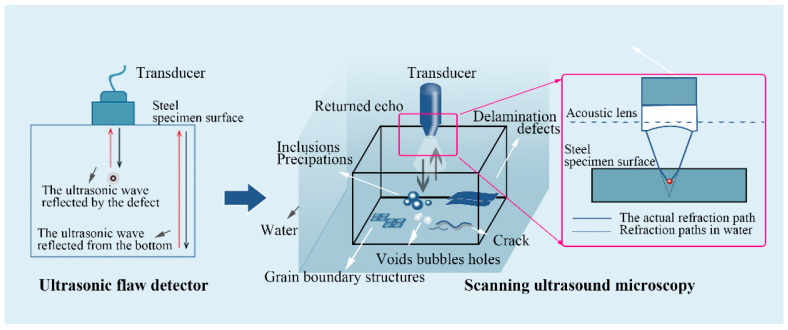
Testing principle of the ultrasonic instrument.

The probe size of the digital ultrasonic flaw detector (HY-CT350, Huayi, Beijing, China) is 13 mm × 13 mm, with a frequency of 2.5 MHz and a maximum longitudinal beam path of 6100 mm. The location of macroscopic defects in the sample is roughly measured, which provides a reasonable scanning region for further determining the exact position of defects in the next stage. All the location defects identified by the preliminary survey are marked. According to the working principle of the UFD shown in Figure 2, it is known that X and Y are coordinates of the defect [25]. Since the ultrasonic wave propagates along the vertical direction in the scanning material, Z can be obtained from the following equation:

(1) $$h=\frac{s\;\times \;c}{2}$$

where *h* is the depth of the defect distance from the upper surface in the unit of mm; *s* is the time in the unit of ns; and *c* is the speed of sound in the steel with a value of 5900 m/s.

The preliminary position of the defect can be determined and marked based on the testing result of the hand-held ultrasonic detector. The edge profile obtained by ultrasonic scanning is considered as the size of the inclusions. A more precise specimen was machined based on the preliminary position to meet the need for high-frequency ultrasonic microscope detection [26]. The top and bottom surfaces of the samples are ground to reduce the surface roughness, and the samples with uniform thickness and flat surface are prepared, so that the influence of the roughness on the ultrasonic echo is minimized [27].

The scanning ultrasonic microscope can examine the inside of a material without damaging the inspection kit. There are two operation modes: one based on ultrasonic reflection and the other based on transmission. The reflection mode, the main working mode, is characterized by high resolution and no limitation on the thickness of the sample, which can effectively detect the inclusions, lattice structure [28,29], internal cracks, lamination defects, holes, bubbles, voids, and other defects in the material [30]. The transducer, as the core of a scanning ultrasound microscope, converts electrical impulses into acoustic pulses, and the sound waves are focused on the sample by an acoustic lens through a coupling agent (usually water, which prevents the sound waves from falling too far in the air) after leaving the transducer. During the experiment, the sample was placed flat in the water tank to keep the surface of the sample under test smooth and horizontal. When the ultrasonic wave contacts the water, the sample surface will be refracted; then, it is re-focused inside the sample. When the ultrasonic wave passes through the defects such as a void, it will be reflected, and then the transducer will receive the ultrasonic echo and the electrical pulse. The water-immersed longitudinal pulse-echo method is adopted to detect the defects in the steel samples at the present experiment. The transducer excitation load is 60 ns pressure load, the wavelength of the longitudinal wave in the steel is 0.12 mm, and the time step is 0.2 ns. By adjusting the frequency and height of the probe, the focus of the transducer is set on the upper surface of the material to ensure the maximum resolution and sound pressure strength for inclusions detection. A probe with appropriate frequency is selected to scan the entire material interior to obtain the whole C-scan imaging of the inclusions inside the material, and then the target area to be analyzed is found in the C-scan image, followed by an accurate scan with a small step value, and finally, the full-wave data is saved.

### 2.3. Inclusions Analysis of Automated Inclusion Analyzer

Some research work has been carried out through SEM (Phenom Scientific, Shanghai, China) in combination with EDS measurements to analyze inclusions in samples. SEM was used to verify and analyze the specific components of the inclusions. However, to verify and compare the inclusions distribution results of ultrasonic detection, the Oxford INCA System (2010 INCA, 2010, Oxford Instruments, Oxford, England) was used in this study to statistically verify the inclusions distribution. It is an integrated SEM and EDS system, which can quickly and accurately provide the composition, size, and quantity of inclusions in the steel.

**Figure 3:**
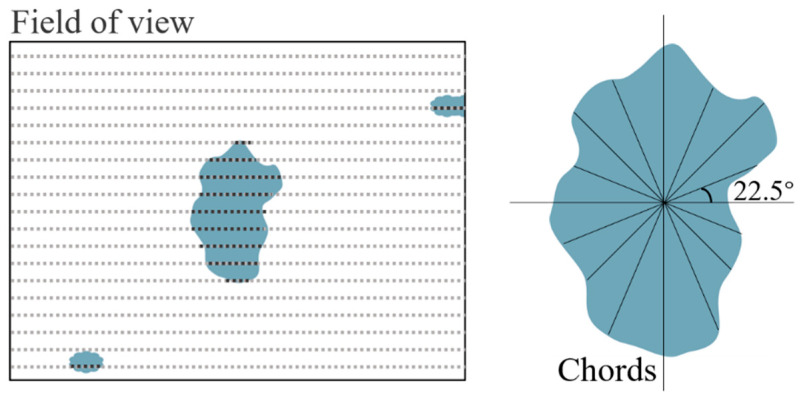
Testing principle of automated inclusion analyzer.

In the Oxford INCA system, the focused electron beam searches the user-specified area. After the selected region is determined, the stepwise scan analysis is carried out first. When the electronic beam detects the inclusions, the geometric center of the inclusions is obtained by using high magnification and a small step moving scan. Then, starting from the geometric center, the scan is carried out in eight directions outward to obtain eight chords of different lengths. According to these chords, the shape and size of the inclusion can be determined, and the parameters such as maximum diameter, minimum diameter, average diameter, and length–width ratio can be obtained. This analysis system recognized inclusions by means of a backscattered electron detector with the help of contrast differences of grayscale thresholding whose equivalent diameter is larger than 0.6 μm between inclusions and the steel matrix. The chemical composition of each particle was recorded by EDS (20 kV) in the automated inclusion analyzer (Oxford INCA EVO18, ZEISS, Oberkochen, Germany). The chemical composition of the geometric center of the inclusion is taken as the composition of the whole inclusion, so the voids and cracks cannot be distinguished. Figure 3 is a schematic diagram of the working principle of the automatic inclusions analyzer.

## 3. Results

### 3.1. Detection Results of the Ultrasonic Experiment

**Figure 4:**
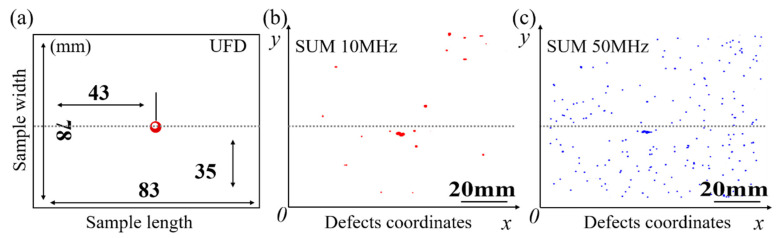
Scanning results of different types of ultrasound methods: (**a**) ultrasonic flaw detector, 2.5 MHz; (**b**) scanning ultrasonic microscope, 10 MHz; (**c**) scanning ultrasonic microscope, 50 MHz.

As shown in Figure 4, with the increase of scanning frequency of the ultrasonic, the distribution information of inclusions on the focus surface becomes more and more comprehensive. The ultrasonic flaw detection instrument with 2.5 MHz scanning frequency in Figure 4a can give a relatively accurate location on large-size defects, but a lot of specific information of micro-defects cannot be identified. Here, we emphasize the large-size defect because it is extremely detrimental to the steel matrix. In combination calculation by Equation (1), it is found that the defect is located on the plane with a depth of 10.88 mm. Based on the location information, the sample is machined to a more precise size by maintaining the large-size defect much closer to the focus plane for ultrasonic testing. SUM (PVA-SAM-300, PVA Tepla AG, Wettenberg, Germany) with a 10 MHz scanning probe is used to determine a more precise location of the defect through scanning the surface of the processed sample (pulse-echo measurement), as results in Figure 4b. High scanning frequency makes the location and contour boundary of defects more specific, but it is still far from the actual shape. The maximum focusing depth of a 10 MHz probe in water was 20.3 mm, which is equivalent to a maximum focusing depth of 5 mm in steel. Correspondingly, the maximum focusing depth of the 50 MHz probe is only 2.5 mm in steel. It is found that the defect is located at the depth of 4.75 mm distance from the top surface, which is still not the optimal focus depth for the 50 MHz probe. After thinning by 3 mm, the target defect is just in the focus plane of the 50 MHz probe, and it is found at a depth of 1.728 mm, as shown in Figure 4c.

**Figure 5:**
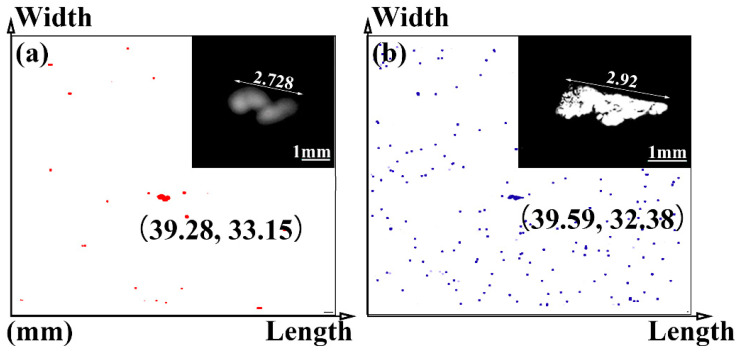
Comparison of size and position information of C-scanning images under different frequencies of an ultrasonic probe of (**a**) 10 MHz; (**b**) 50 MHz.

Table 2 shows the technical parameters of different ultrasonic probes. It is known that a cylindrical focusing area is formed during the scanning process of the ultrasonic probe, and the length of the cylindrical area, namely the focal length, is dependent on media, which changes from water to steel. When the focusing depth of the probe is 1.728 mm, the maximum size of inclusions detected is 2.92 mm, as shown in Figure 5.

The background noise is inevitable in the process of ultrasonic signal transmission and amplification during the material detection. A large gain produces a high-level noise. This indicates that the larger the defect size, the larger the echo amplitude at a certain depth. Thus, the specific size of the inclusions cannot be determined only by the amplitude of the scanning echo. In addition, the defect’s size as shown in Figure 5 obtained by binary processing could have a certain deviation from the actual size considering the unclear edge contour in the result. The actual morphology of the defect needs to be verified through in situ dissection observation in combination with SEM or EDS, which will be discussed in the subsequent section.

### 3.2. Quantitative Defect Distribution by SUM

**Figure 6:**
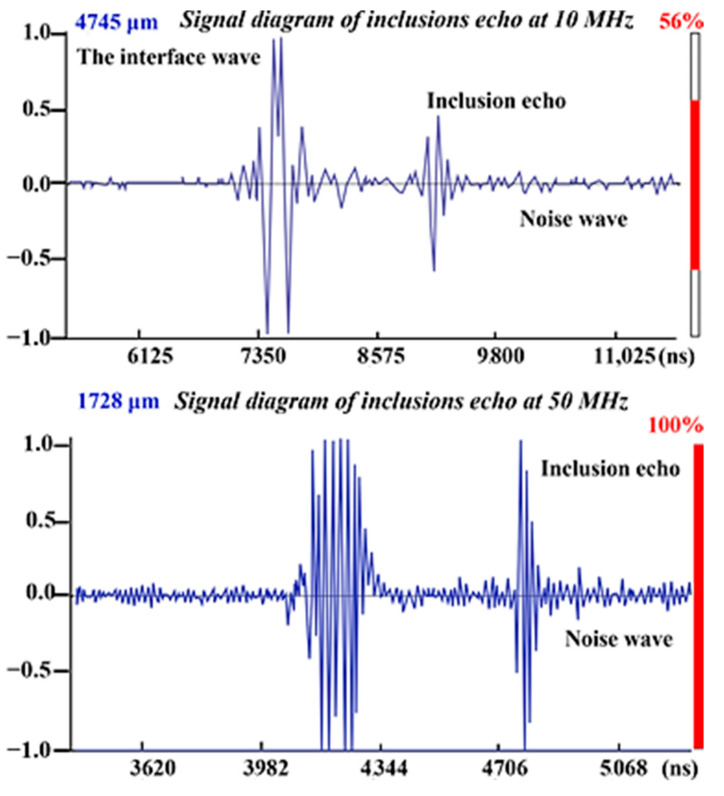
A longitudinal wave scanning signal of the largest inclusion under different frequency probes (10 MHz and 50 MHz).

Figure 6 shows the signal waves obtained by scanning the largest inclusion. The amplitude within (*μ* − 3*σ*, *μ* + 3*σ*) is the noise signal, and the amplitude outside is the inclusion echo signal. The selected place without inclusions was detected, and then the interface wave and the bottom wave were removed. The mean value of the signal in the middle region was *μ*, and *σ* was the variance [31]. It can be seen from Figure 6 that the return amplitude of the inclusions of the target defect is relatively high, and its position is on the depth surface of 4.75 mm and 1.728 mm below the upper surface.

When the 50 MHz probe was used to detect the target defect, the defect was located at a depth of 1.728 mm. In the ultrasonic image, there are many points below 50 μm, and it is difficult to extract the edges due to the large number and small size, so only the inclusions above 50 μm were counted. The statistical results are shown in Table 3.

**Figure 7:**
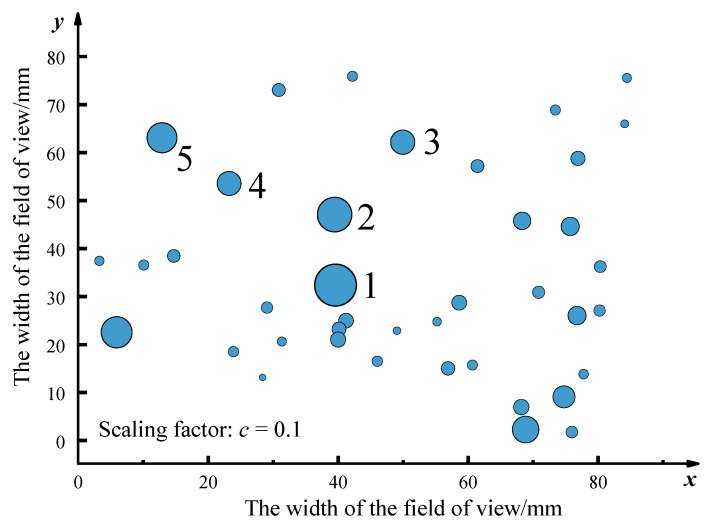
Defect size statistics and distribution.

The size and the coordinate position of the distribution of 40 defects were statistically analyzed, and the results are shown in Figure 7. According to the analysis of the test results, the defects near the largest defects are not only large but also densely distributed.

### 3.3. Characteristic Comparison of Inclusions-Induced Defects by Ultrasonic and Scanning Electron Microscopy

To further detect the characterizations of the defects, the samples were dissected along the top surface of the defects; five 10 mm × 10 mm areas were selected, which respectively contained the five largest defects. The sample was dissected layer by layer by the precision machining grinder according to the test results. After the sample was processed to the depth of the target defect, the sample was dissected at the thickness of 0.1 mm per layer and observed alternately with the electron microscope.

Table 4 shows the detection results of five major defects by scanning ultrasonic microscope and scanning electron microscope. According to the results of scanning electron microscopy, it can be seen that many kinds of internal defects existed. The target defect is the crack generated by the composite inclusion of Al_2_O_3_, MgO, and CaO, whose size is more than 200 μm. In addition, the length of the target defect detected by the scanning ultrasound microscope is 2.92 mm, while the size of it just is 0.76 mm under the scanning electron microscope. Since the ultrasonic water immersion test measured the outermost edge of the inclusions, and the section obtained in the sample preparation before the electron microscope scanning was usually not the maximum section of the inclusions, the actual size obtained by the electron microscope scanning was smaller than that obtained before.

**Figure 8:**
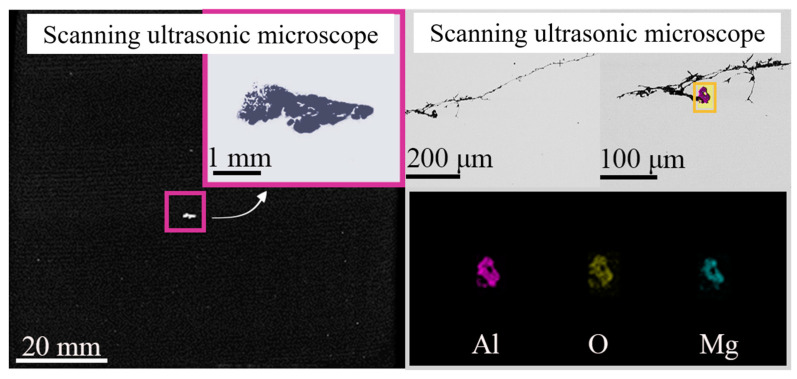
Electron microscope images and elements mapping after dissection of the maximum defect. The large inclusions on the scanning surface were statistically analyzed, and the number, types, and average particle size of inclusions were summarized, as shown in Figure 9. The number of inclusions of Al_2_O_3_-SiO_2_ type is the largest, while the average particle size of the inclusions of Al_2_O_3_-CaO-MgO and Al_2_O_3_-MnS types is larger: both over 20 μm.

Figure 8 is the elemental mapping of the target defect. The defect is caused by a large size brittle Al_2_O_3_-MgO inclusion, which was detrimental to the fatigue life of gear steel, especially under high load conditions due to its poor plastic deformation ability and high hardness. During the rolling process, the brittle Al_2_O_3_-MgO inclusions are more likely to be broken along the rolling direction, and the cracks, holes, and other micro defects around the inclusions are easily formed, which caused damage [32].

### 3.4. Distribution of Inclusions Based on Automated Inclusion Analyzer

To further determine the distribution of inclusions and compare it with SUM, another five 10 mm × 10 mm samples were taken on the same surface for automatic inclusions analysis, and each sample was analyzed with six fields of view.

**Figure 10:**
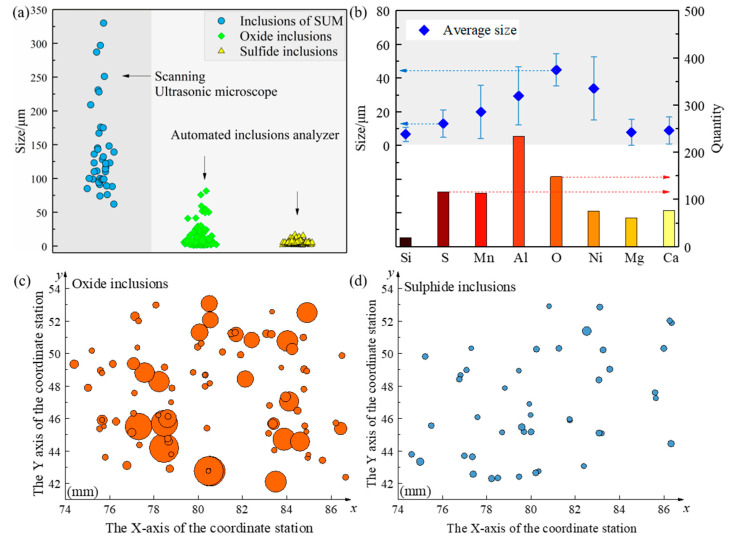
The comparison of inclusions size between automatic inclusions analysis and scanning ultrasound microscopy (SUM) analysis (**a**) and the average size and quantity in different inclusions statistics (**b**), and the planar distribution (**c**,**d**) of inclusions with different compositions were also made.

As shown in Figure 10a, the size of the inclusions under the scanning ultrasound microscope is more than 50 μm, while the size of the inclusions detected under the automated inclusions scanning analyzer is in the range of 1 to 100 μm. Oxide inclusions are slightly larger, with the largest inclusions around 100 μm, while sulfide inclusions range in size from 1 to 30 μm. The echo of ultrasonic nondestructive testing can obtain the utmost profile of the larger inclusions in the sample at different depths, while the cross-section of the inclusions in a certain layer can be obtained through the scan of the automatic inclusion analyzer. Scanning ultrasound microscope detects large inclusions in the whole sample range. Figure 10a shows the size statistics of sulfide inclusions and oxide inclusions in a specific field of view for automatic inclusion analysis. It can be seen that the size detected by ultrasonic detection microscope is three to four times that detected by automated inclusion analyzer. Different from the results of ultrasonic microscopic detection and scanning, the inclusion automatic analysis instrument can not only obtain the size and distribution of inclusions but also obtain the composition of different inclusions, as shown in Figure 10b. In this field of view, the average size of oxide inclusions is 42 μm, and the average size of sulfide inclusions is 7 μm. Figure 10c,d show the size and distribution spacing of oxides and sulfides in this field of view. Compared with sulfide, oxides have a larger size and uneven size and distribution. Different from Figure 7, the position coordinates obtained by ultrasonic detection are based on the real coordinates of the sample itself. The automatic analyzer of inclusions, from the perspective of the coordinate, is relative to the sample’s given relative distance; it can only see the length and distribution of inclusions spacing. If cannot be combined with sample’s placement for conversion, it actually cannot obtain the inclusions of real location information.

## 4. Discussion

### 4.1. Extrapolation of the Log-Normal Function on Inclusions in Steel Based on Statistical Method

The grain size distribution in many metals and alloys is logarithmic, and most inclusions in steel also have a logarithmic normal distribution in terms of size. The size of the inclusions is usually expressed in terms of equivalent diameter (*d*), which is the diameter of a circle with the same area as the inclusions or the diameter of a sphere with the same volume [33].

It is assumed that the size distribution of inclusions in steel conforms to the lognormal distribution, that is, the frequency (ln*d*) of the logarithm of grain size conforms to the normal distribution [34]. The probability density function of inclusion of size *y* = *d* can be calculated by Equation (2):

(2) $$f\left(x\right)=\frac{1}{\sigma \sqrt{2\pi }}\exp\left(-\frac{{\left(x-\mu \right)}^{2}}{2\sigma^{2}}\right),\;-\infty <x<\infty$$

where *x* = ln(*y*) and σ and μ are the standard deviation and mean of *x*. Then, *y* = exp(*x*) has a logarithmic normal distribution:

(3) $$f\left(y\right)=\frac{1}{y\sigma \sqrt{2\pi }}\exp\left(-\frac{{\left(lny-\mu \right)}^{2}}{2\sigma^{2}}\right),\;y>0$$

**Figure 11:**
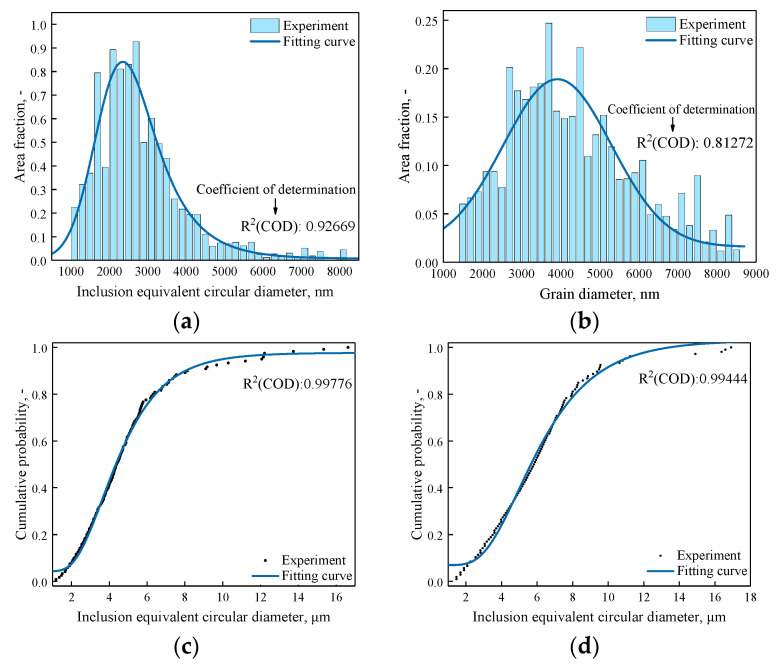
Logarithmic normal distribution and cumulative distribution function of sulfide inclusions and oxide inclusions (**a**) Logarithmic normal distribution of sulfide inclusions; (**b**) Logarithmic normal distribution of oxide inclusions; (**c**) Cumulative distribution function of sulfide inclusions; (**d**) Cumulative distribution function of oxide inclusions.

In the statistical process of inclusions size distribution, the distribution probability of inclusions in the plane is related to the field area and the area of inclusions, and the product of the occurrence times and area fraction of inclusion of a certain size is taken as its distribution probability. Figure 11 shows the logarithmic normal distribution fitting and cumulative distribution function of sulfide inclusions and oxide inclusions in the steel. The determination coefficient R^2^ value is the multiple decision coefficient of the fitting function, which is used to evaluate the fitting effect and has a range between 0 and 1. The R^2^ values at each position are close to 1, and the fitted curve model was quite reasonable. As evaluated by the R^2^ values, the sizes and number distributions of inclusions kept a good fitness with the curve models. It can be seen from Figure 11 that sulfide inclusions and oxide inclusions in steel conform to lognormal distribution, and the proportion of inclusions decreases gradually with the increase of the size of inclusions. According to the shape of the fitting curve, it can be seen that in the same kind of steel, the probability of finding large oxide inclusions is higher than that of sulfide inclusions.

According to the inclusion distribution curve obtained by fitting, the size distribution interval of different inclusions in steel can be predicted to a certain extent, and the size range of the maximum inclusions increases with the increase of the steel volume fraction. The standard method of fitting logarithmic normal distribution to predict the size of inclusions in steel requires quantitative measurement and statistics of the size of inclusions within a defined range. However, this requires a lot of precise data, and many smaller inclusions cannot be detected by instruments. Other simpler methods are needed to predict the maximum defects in steel.

### 4.2. Largest Extreme Value Distribution (LEVD)

In essence, the theory of extreme statistical analysis is extrapolation, which can accurately estimate the distribution of the maximum value at the end according to the independent data of a certain number of random fields of view. This method can be used to avoid the defect that the instrument is not accurate enough to measure very small inclusions. In the LEVD analysis method, only the maximum inclusion in the randomly selected region should be measured and counted.

**Figure 12:**
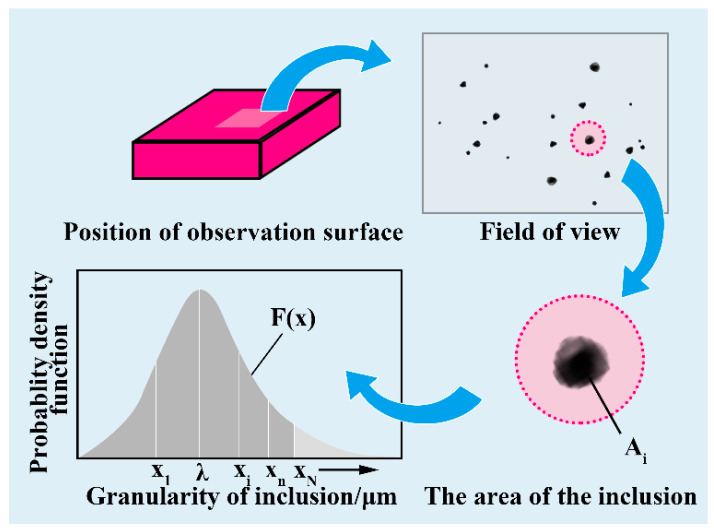
Schematic diagram of data fitting and sampling observation by LEVD.

Assuming that there are tens of thousands of inclusions in the total sample, n sub-samples are randomly selected in this population area; then, the maximum particle size of inclusions in each sub-sample (X_1_, X_2_…X_n_) conforms to the distribution of the Gumbel approximation function (as shown in Figure 12, the area of each sample is A_N_). The LEVD has the following probability density function [35]:

(4) $$g\left(x\right)=\frac{1}{\delta }exp\left\{-\left[\left(\frac{x-\lambda }{\delta }\right)+exp\left(-\frac{x-\lambda }{\delta }\right)\right]\right\}$$

and cumulative probability function:

(5) $$G\left(x\right)=exp\left\{-exp\left[-\frac{\left(x-\lambda \right)}{\delta }\right]\right\}$$

where *λ* and *δ* are referred to as the location and scale parameters, respectively.

Define a standard field of view and calculate the area A of the maximum inclusion in each standard field of view. Let *x_i_* = √area be the size of the *i*-th defect in the sample of collected data (*i* runs over the *n* defects of the sample sorted in ascending order). The cumulative probability of the *i*-th defect size not greater than *x_i_* is:

(6) $$G\left(x_{i}\right)=\frac{1}{\left(N+1\right)}$$

After the estimated value ($\hat{\lambda }$, $\hat{\delta }$) of parameter *λ* and *δ* is obtained, the maximum inclusion size value *x_max_* can be calculated under probability *G(x)* by Equation (7):

(7) $$x_{max}=\delta \left[-ln\left(G\left(x\right)\right)\right]+\lambda$$

Define *y* as the standardized variable:

(8) $$y=-ln\left(-\ln G\left(x\right)\right).$$

The *λ* and *δ* of the formula can be calculated from the measured value of the maximum particle size of each sub-sample and the corresponding *y*, and *y* is given by the corresponding *G(x)* in terms of Equation (8). Combined with Equation (7), the maximum particle size *(x_max_*) of the whole population can be obtained.

**Figure 13:**
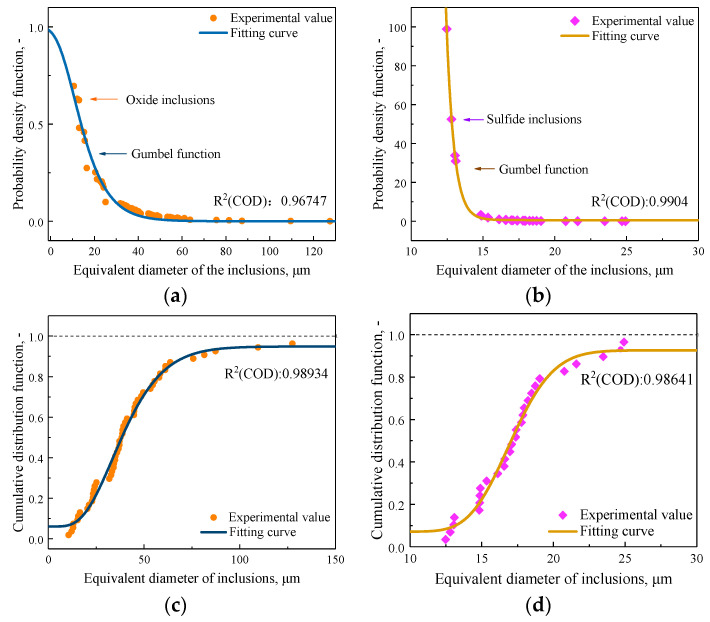
Probability density function and cumulative distribution function curves of the maximum of oxide inclusions (**a**,**c**) and sulfide inclusions (**b**,**d**) in different fields of view.

The maximum particle sizes of five samples with a total of 30 viewing fields were estimated by using the LEVD statistics method, and the maximum inclusion particle sizes under the cumulative distribution of different Gumbel distributions were calculated. Figure 13 shows the probability density function and cumulative distribution curve of the maximum oxide inclusions and sulfide inclusions in different fields of view.

**Figure 14:**
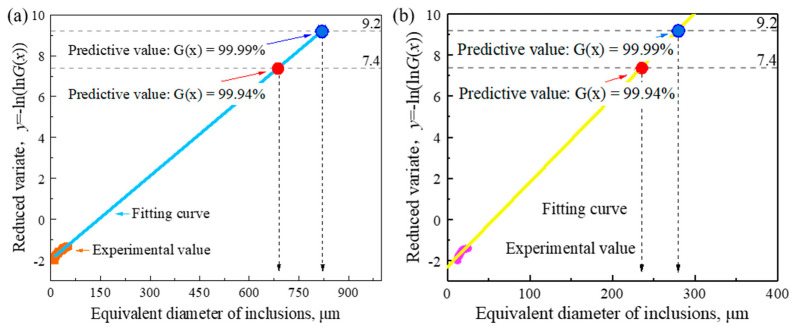
The maximum size of oxide inclusions (**a**) and sulfide inclusions (**b**) inferred by the LEVD method.

The predicted value of the extreme value distribution is related to the total size of the sample. Generally, the larger the sample, the higher the predicted value will be. The drawing results are shown in Figure 14. According to the analysis results, when the cumulative distribution function *G*(*x*) is 99.99% [36], the predicted largest oxide inclusion is about 820 μm, and the predicted sulfide oxide inclusion is about 275 μm. When the cumulative distribution function *G*(*x*) is 99.94% (there are 99% viewing fields), the predicted largest oxide inclusion is about 690 μm, and the predicted sulfide oxide inclusion is about 235 μm.

For the largest defect in the sample, the size of the inclusion was 2920 μm when detected by water-immersion coupled ultrasonic microscopy, the size of the inclusion was 760 μm when accurately located by scanning electron microscopy, and the size of the inclusion was calculated by LEVD analysis as 860 μm. According to the actual detection results of SEM, when the cumulative distribution function *G(x)* is 99.94%, the inferred results are closer to the results of SEM. According to the SEM results given above, the largest inclusions are cracks caused by magnesium and aluminum oxides. However, in the LEVD method, the size we obtained is the size of the oxide itself, in which it can be inferred that cracks can already occur when the inclusions reach 690 μm. Ultrasound non-destructive testing showed that the outermost edge, containing the entire crack, was three to four times the size of the automated inclusion analyzer.

The analysis shows that ultrasonic microscopy can detect the defects at a certain depth inside the sample without destroying the sample, the larger defects will not be missed, and the detected size is also the largest. Scanning electron microscope analysis has randomness in field of view selection, but combined with the ultrasonic microscope, inclusions can be accurately located, and defect size and composition can be detected more completely under the condition of strict control of sample preparation. Unlike the former two, the inclusions size inferred by the LEVD analysis method is the possible original size of the largest inclusion, excluding the cracks produced later. Therefore, in the case of sufficient data volume, the maximum value of different types of inclusions can be completely inferred according to the LEVD analysis method, and even the size critical range of further derived defects can be judged by this method. Through the comparison of the three methods, we can get that the inclusion analysis in a certain number of small fields on a random plane, combined with the analysis of the inclusion in ultrasonic detection on a different depth plane, and we can evaluate the production quality of the whole metal material. In this way, a small number of samples can be taken for prediction evaluation without damaging the whole metal, but this method needs to be corrected and verified by a large number of experiments in the future to correct this method and specific parameters.

## 5. Conclusions

(1) The detection system that can accurately characterize the non-metallic inclusions in steel was determined by experiments. The determination of detection depth, frequency, and other parameters is conducive to the accurate location of inclusions and dissection.

(2) The proposed integrated approach with destructive and non-destructive methods can show the in situ information of inclusions as well as give the possible relationship between inclusions and process and material properties; the analysis speed is faster, and it can accurately identify the shrinkage cavities, cracks, and other defects in steel.

(3) Oxide inclusions and sulfide inclusions in steel are following the log-normal distribution in size, and the maximum inclusions in each sample obey the Gumbel extreme distribution. The maximum size of inclusion detected by SUM is 2920 μm, and the size detected by SEM is 860 μm. When the cumulative distribution function *G(x)* is 99.94%, the inferred result is 690 μm, which is closer to the SEM value. The maximum size inclusion in the sample was deduced and verified by LEVD analysis. The predicted value is in very good agreement with the size of the largest inclusions detected by SEM. The results show that this method can be used to infer the size of a certain type of maximum inclusion in a certain area under the condition of sufficient data.

(4) There are still some limitations in this method, such as the failure to provide accurate three-dimensional morphology, particle size distribution, and other information of the target inclusions; the ultrasonic detection is not suitable for the observation of small defects and inclusions; and the accuracy of the anatomical operation is strictly required. Therefore, in the comprehensive characterization of inclusions, different methods can be selected according to different needs to guide the improvement of steel performance more effectively.

## Figures and Tables

**Figure 9:**
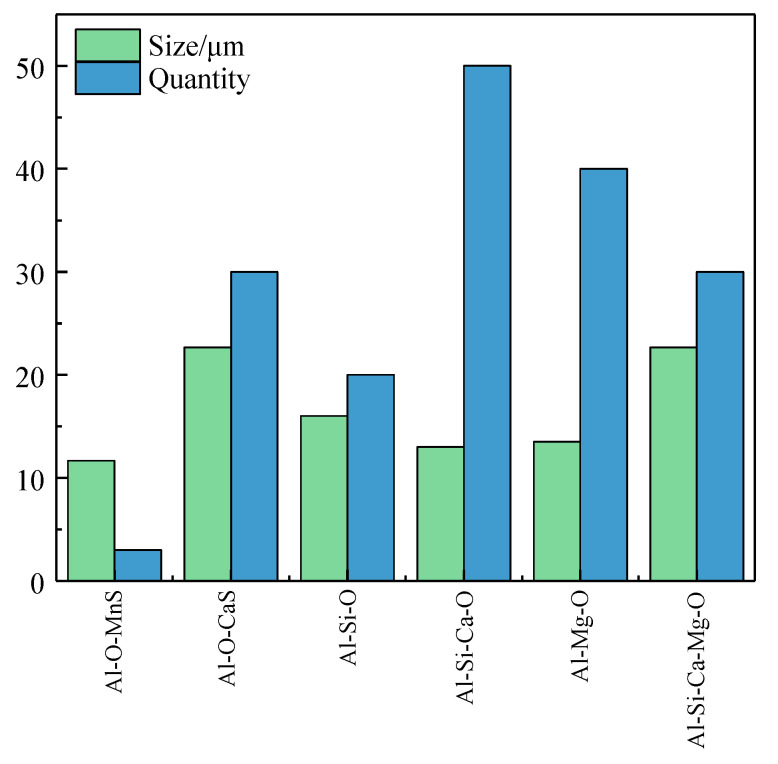
The number and the average size of inclusions of different types.

**Table 1:** Chemical composition of the investigated steel (wt %).

Elements	C	Si	Mn	P	S	Cr	Ni	Mo
Content	0.15–0.21	0.40	0.5–0.9	<0.035	<0.035	1.5–1.8	1.40–1.70	0.25–0.35

**Table 2:** Technical parameters of different ultrasonic probes.

Parameter	Ultrasonic Flaw Detector	Scanning Ultrasonic Microscope	
Frequency/MHz	2.5	10	50
Focal length in water/mm	-	20.3	10
Focal length in steel/mm	-	5	2.5
Length of focal column/mm	-	0.68	0.3
Depth of focus/mm	10.88	4.75	1.728
Imaging depth range/mm	-	4.41–5.09	1.578–1.878
Scan area	78 mm × 83 mm	78 mm × 83 mm	78 mm × 83 mm

**Table 3:** Statistics of defect dimensions in 18CrNiMo7-6 steel.

Defect size	2920 μm	300–400 μm	200–300 μm	100–200 μm	50–100 μm
Quantity	1	1	6	20	12

**Table 4:** Comparison of ultrasonic microscopic scanning and electron microscope scanning images.

	Scanning Ultrasonic Microscope	Scanning Electron Microscope	Elements
1	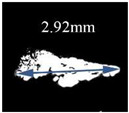	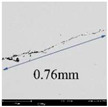	Al-O-Mg
2	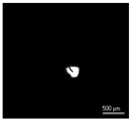	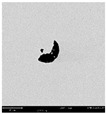	Al-O-Mg-Ca
3	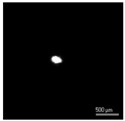	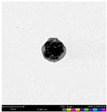	Al-O-S-Ca
4	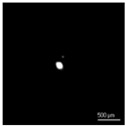	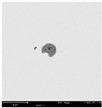	Al-O-Mg-Ca
5	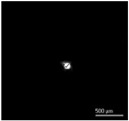	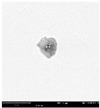	Al-O-S-Mg

## Data Availability

The data presented in this study are available on request from the corresponding author.
